# Complete genome sequence of *Agrobacterium pusense* Bbcg2-2, a strain isolated from blueberry crown gall in Taiwan

**DOI:** 10.1128/mra.01083-23

**Published:** 2024-01-08

**Authors:** Ru-Bin Yao, Xiao-Hua Yan, Hsi-Ching Yen, Shin Lee, Chia-Ching Chu, Cheng-Fang Hong, Chih-Horng Kuo

**Affiliations:** 1 Institute of Plant and Microbial Biology, Academia Sinica, Taipei, Taiwan; 2 Department of Plant Pathology, National Chung Hsing University, Taichung, Taiwan; 3 Biotechnology Center, National Chung Hsing University, Taichung, Taiwan; The University of Arizona, Tucson, USA

**Keywords:** genome, *Agrobacterium*, *Agrobacterium pusense*

## Abstract

*Agrobacterium pusense* Bbcg2-2 is a strain isolated from a crown gall sample of blueberry (*Vaccinium corymbosum*) cultivar “Flicker” grown in Taiwan. The complete genome sequence of this bacterium consists of a 2,798,342-bp circular chromosome, a 2,140,031-bp linear chromid, and a 386,016-bp circular plasmid.

## ANNOUNCEMENT

The genus *Agrobacterium* contains soil-dwelling bacteria that are often associated with plants (1). Some strains harbor tumor-inducing plasmids and are capable of inducing abnormal tissue proliferation in the infected plants (2, 3). To better characterize the natural diversity of these bacteria, we isolated a novel strain Bbcg2-2 from a crown gall sample of blueberry (*Vaccinium corymbosum*) cultivar “Flicker” and determined its complete genome sequence. Unless stated otherwise, the methods were based on the cited references; the kits were used according to the manufacturer’s instructions; and the bioinformatics tools were used with the default settings.

The crown gall sample was collected in a blueberry farm (Wufeng District, Taichung City, Taiwan; GPS coordinates: 24.0549 N, 120.6803 E) on 17 February 2023. The strain isolation protocol was based on that described for dicot samples (4). Briefly, the sample was surface sterilized with 10% bleach, washed thrice with sterilized distilled water, then finely ground and immersed in sterilized distilled water. After incubation overnight at room temperature and the presence of motile rod-shaped bacteria was confirmed via microscopy, the suspension was streaked on Clark’s selective medium for purification of single colonies through three iterations (5).

For DNA extraction, a single colony was picked and grown in 1-mL liquid 523 medium (6) at 28°C for 20 hours then processed using Nanobind CBB kit (PacBio, USA). For Illumina sequencing, the library was prepared using KAPA LTP Library Preparation Kit (Roche, Switzerland) and sequenced on a NovaSeq 6000 to generate 32,205,577 pairs of 150-bp reads. For Oxford Nanopore Technologies (ONT) sequencing, the library was prepared using ONT Ligation Sequencing Kit (SQK-LSK109) and sequenced on a MinION (R10.4.1, FLO-MIN114). Basecalling was performed using Guppy v.6.5.7 with the super accuracy mode, producing 25,901 reads (combined: 426,981,262 bp, N50: 34,343 bp). All reads were assembled using Unicycler v.0.4.9b (7) without pre-processing. For validation, the Illumina and ONT reads were mapped to the assembly using BWA v.0.7.17 (8) and Minimap2 v.2.15 (9), respectively. The mapping results were programmatically checked using SAMtools v.1.9 (10) and visually inspected using IGV v.2.16.1 (11).

The complete genome sequence of this bacterium consists of a 2,798,342-bp circular chromosome (59.3% G + C), a 2,140,031-bp linear chromid (59.2% G + C), and a 386,016-bp circular plasmid (57.7% G + C). The Illumina and ONT reads provided 1,813.1- and 75.3-fold coverage, respectively. Annotation by PGAP v.6.6 (12) identified 12 rRNA genes, 54 tRNA genes, 4,912 protein-coding genes, and 71 pseudogenes. Comparisons with *Agrobacterium* genome sequences available from GenBank (as of 28 August 2023) using FastANI v.1.1 (13) found that this newly isolated strain Bbcg2-2 shares 98.19% genome-wide average nucleotide identity with the type strain of *Agrobacterium pusense* (LMG 25623, NCBI assembly accession GCA_900102105.1).

## Data Availability

This genome project was deposited in National Center for Biotechnology Information (NCBI) under accession number PRJNA1010975. The raw reads were deposited at the NCBI Sequence Read Archive under accession numbers SRX21543989–SRX21543992. The genome sequence was deposited at GenBank under accession numbers CP133673–CP133675.
